# Analysis of Kinect-Based Human Motion Capture Accuracy Using Skeletal Cosine Similarity Metrics

**DOI:** 10.3390/s25041047

**Published:** 2025-02-10

**Authors:** Wenchuan Jia, Hanyang Wang, Qi Chen, Tianxu Bao, Yi Sun

**Affiliations:** School of Mechatronic Engineering and Automation, Shanghai University, Shanghai 200444, China; lovvchris@shu.edu.cn (W.J.); why20001013@shu.edu.cn (H.W.); chenqi1125@shu.edu.cn (Q.C.); baotianxu@shu.edu.cn (T.B.)

**Keywords:** motion capture, Azure Kinect DK, body posture, recognition accuracy, cosine similarity

## Abstract

Kinect, with its intrinsic and accessible human motion capture capabilities, found widespread application in real-world scenarios such as rehabilitation therapy and robot control. Consequently, a thorough analysis of its previously under-examined motion capture accuracy is of paramount importance to mitigate the risks potentially arising from recognition errors in practical applications. This study employs a high-precision, marker-based motion capture system to generate ground truth human pose data, enabling an evaluation of Azure Kinect’s performance across a spectrum of tasks, which include both static postures and dynamic movement behaviors. Specifically, the cosine similarity for skeletal representation is employed to assess pose estimation accuracy from an application-centric perspective. Experimental results reveal that factors such as the subject’s distance and orientation relative to the Kinect, as well as self-occlusion, exert a significant influence on the fidelity of Azure Kinect’s human posture recognition. Optimal testing recommendations are derived based on the observed trends. Furthermore, a linear fitting analysis between the ground truth data and Azure Kinect’s output suggests the potential for performance optimization under specific conditions. This research provides valuable insights for the informed deployment of Kinect in applications demanding high-precision motion recognition.

## 1. Introduction

The continuous advancement of motion capture technology propelled its application far beyond the realms of film production and game design, with notable adoption in domains such as medical rehabilitation, virtual reality, and robot control [1,2,3]. In particular, markerless vision-based motion capture devices, owing to their low hardware costs and ease of use [4], demonstrate a broader application potential compared to traditional marker-based systems [5]. Undoubtedly, Microsoft’s Azure Kinect DK series stands as a representative example of markerless motion capture devices [6], distinguished not only by its compact design and open software framework, but also by its superior image modulation frequency, depth data accuracy, and its inherent human skeletal joint recognition capabilities [7,8]. However, as is characteristic of markerless motion capture devices, the correctness and precision of its recognition are susceptible to factors such as illumination variations and occlusions in Kinect’s field of view.

In the film industry, motion capture data are typically acquired offline, allowing for the mitigation of data deviations through iterative acquisition or heuristic adjustments based on experience and intuition. In interactive gaming, motion capture data are processed in real-time, but inaccuracies often remain unnoticed unless they manifest as egregious errors. However, in real-world applications, such as medical rehabilitation, the implications of motion capture data deviations become critical and can no longer be disregarded, as they directly influence the effectiveness of the application and potentially introduce significant risks. For Kinect, as the applications increasingly diversify and become more practical, a rigorous evaluation of its accuracy becomes ever more paramount.

For instance, Amprimo et al. employed the Azure Kinect for the remote assessment, detection, and rehabilitation of Parkinson’s disease patients [9]. This same sensor has also been utilized for classifying and detecting individuals with depression [10] and for monitoring geriatric clinical conditions [11], collectively demonstrating its popularity in clinical healthcare. Bärligea et al. leveraged the Azure Kinect as a motion-tracking signal generator for weightlessness simulation, showcasing its potential in manned space mission planning and other aerospace applications [12]. By integrating the Kinect with other sensing devices and control techniques, its range of applications can be further expanded. Examples include combining it with robot models to achieve collision-free human–robot interaction [13], integrating it with IMUs for more stable human motion measurement [14], and introducing deep learning techniques to enable upper limb functional assessment using a single Kinect v2 sensor [15].

Various methods have been proposed to enhance Kinect’s motion capture accuracy. For instance, a Support Vector Machine (SVM) classifier was employed to improve the accuracy of human action recognition [16]. Park et al. designed a whole-body driven scanner consisting of three Kinect v2 sensors, mitigating the time cost and privacy concerns associated with wearing tight-fitting clothing in traditional 3D whole-body scanning while achieving high prediction accuracy [17].

Since the parallel use of Kinect sensors can enhance accuracy, various multi-sensor approaches have been investigated. One study proposed using two Kinect sensors to acquire upper limb joint angle data from different perspectives, resulting in significantly improved accuracy and robustness of joint angle trajectory recognition [18]. Another study combined data from multiple Azure Kinect sensors, transforming depth information into 3D positions to mitigate occlusion issues inherent in single-camera setups [19]. A data fusion algorithm for three Kinect devices was developed to enhance the accuracy of human skeletal tracking [20]. Furthermore, a spatiotemporal calibration method for multiple cameras was proposed to maximize coverage of the captured subject and minimize occlusions, leading to a substantial improvement in the accuracy of Azure Kinect motion capture [21].

However, considering the practical advantages of deploying a single Kinect and that using multiple Kinect sensors somewhat contradicts the original intent of its ease of use, there has been a growing body of research focusing on the motion capture performance of a single Kinect. Yeunga et al. compared the accuracy of gait tracking using Azure Kinect, Kinect v2, and Orbbec Astra across five camera viewpoints, demonstrating the superior performance of Azure Kinect in tracking hip and knee joints in the sagittal plane [22]. Bilesan discussed how to use inverse kinematics to more accurately convert spatial node data obtained from a single Kinect into joint angle data [23] and evaluated its effectiveness using a real robot [24]. Beshara, through clinical experiments based on multiple testing devices, demonstrated that Kinect exhibits high reliability in shoulder range of motion (ROM) measurements [25]. The depth and spatial accuracy of the Azure Kinect DK have also been compared with those of its predecessors, Kinect v1 and Kinect v2, further validating its advantages in 3D scanning applications [6,26]. Büker et al. evaluated the repeatability of the Azure Kinect, analyzing the spatiotemporal progression and differences in derived parameters from 100 body tracking experiments, revealing significant variations in joint positions under different processing modes and thus recommending careful selection of processing modes and consistent use of the same computer hardware for all analyses in practical applications [27].

Evidently, even the latest Azure Kinect DK device does not yet represent a sufficiently precise motion capture apparatus. Nevertheless, considering the significant advantages and continuous improvement [28,29,30,31,32,33,34] of such markerless motion capture systems in practical applications, as evidenced by the research of Martiš [29], Antico [32], and Milosevic [33] et al., which demonstrates that markerless recognition technologies can significantly reduce assessment time in clinical medicine, the advantages and potential of markerless techniques in clinical settings are undeniable when compared to traditional marker-based systems (e.g., Vicon) and methods. This compels us to engage in a deeper consideration of the following question:

(1) What is the precise accuracy of full-body human motion capture using a single Kinect device? Specifically, how can we design a suitable motion capture data accuracy evaluation scheme that considers its potential practical application requirements, thereby providing reference accuracy data and potential guidelines for its application development? It is pertinent to note that existing accuracy analyses and evaluation methods are primarily categorized into two types. The first stems from image analysis techniques, generally emphasizing the spatial position of joint nodes rather than the morphology of the skeletal system itself. The second directly compares joint rotation angles [23,34], while neglecting positional accuracy. While we acknowledge that discussions regarding the fidelity of joint rotation angle tracking have become increasingly important with the advancement of research on human-like motion, the accuracy of joint position remains crucial, particularly in tasks requiring spatial positioning. Therefore, devising a new and appropriate accuracy evaluation method that represents a compromise between these two types of accuracy (joint angle and joint position) is a worthwhile and practical research direction.

(2) Under the current technological constraints of a single Azure Kinect DK device, what is the potential for subsequent improvement and enhancement of its motion capture data? After all, existing accuracy discussions primarily focus on its previous versions, such as Kinect v2.

We contend that these two questions are pivotal for the deeper application of Kinect in the future. However, to the best of our knowledge, there is currently a paucity of comprehensive research in this area; despite their fundamental importance, most studies continue to focus on showcasing the application cases and potential of Kinect in a wider range of fields. Therefore, this paper addresses these two questions to serve as a valuable supplement to the existing research on the accuracy of Kinect.

In this work, we adopt the concept of cosine similarity between vectors to analyze and evaluate the spatial directional consistency between skeletal recognition results and their ground truth. Since this metric is directly related to the spatial position accuracy of the joints and can be further correlated with the accuracy of joint angles, it combines the advantages of both types of accuracy analysis metrics. Based on a cosine similarity analysis of both local body segments and the entire skeleton, our findings reveal that Azure Kinect DK achieves high skeletal recognition accuracy for stationary postures, with a distinguishable decline in accuracy during motion. When the distance and orientation between the human body and the Kinect camera are maintained within a certain range, the Kinect demonstrates good recognition accuracy and stability, while, as these parameters deviate from this range, the stability of recognition decreases significantly, and the accuracy consequently declines. For individual skeletal segments, the accuracy of distal limb segments is generally lower than that of their proximal counterparts, although exceptions occur under specific conditions, which are analyzed in detail in this study. Furthermore, attempts to post-process and correct motion capture data based on the ground truth data suggest that the accuracy of Azure Kinect DK’s motion capture can be further improved under certain conditions. These novel experimental findings provide practical references and recommendations for the in-depth application of this popular device in tasks requiring high-precision motion recognition.

## 2. Materials and Methods

### 2.1. Experimental Setup

Two identical Azure Kinect DK devices (hereinafter referred to as Kinect), model 1880, with color camera firmware version 1.6.11 and depth camera firmware version 1.6.79, were selected for the evaluation tests. It is important to clarify that these devices were not utilized concurrently; rather, one served as a control to assess the generalizability of the acquired data.

Furthermore, considering that marker-based motion capture systems are capable of acquiring high-precision spatial positioning data, a Visualeyez system manufactured by Phoenix Technologies was employed to accurately acquire the spatial pose data of markers and subsequently generate human motion pose data that could serve as ground truth. This device, specifically the Visualeyez III VZ10K PTI 3D Motion Capture system (hereinafter referred to as Visualeyez), is an active marker-based tracking system [35,36]. It features a maximum sampling frequency of 10,000 Hz and a sampling accuracy of 0.1 mm. With a spatial resolution of 15 μm, a square field of view (FOV) of 100°, and a latency of 0.3 ms, it is capable of supporting high-precision dynamic capture [37].

**Figure 1:**
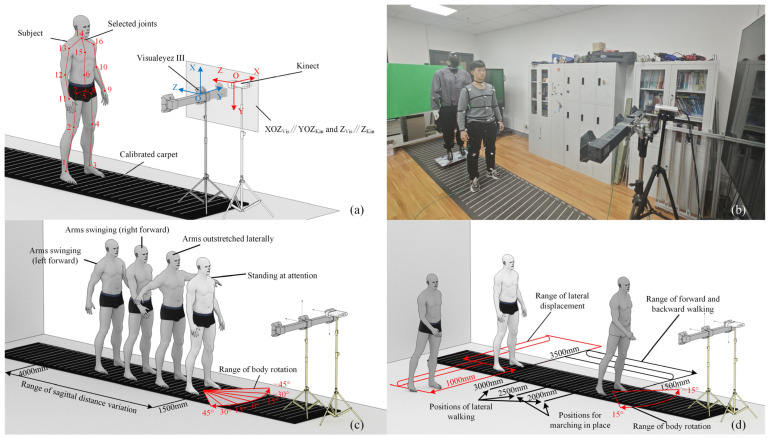
Illustration of the experimental protocol and scenarios. (**a**) Placement of the two types of motion capture devices, with the default coordinate system of Kinect indicated in red and that of Visualeyez in blue. Red nodes and lines superimposed on the human figure are directly generated from skeletal data acquired by Kinect. (**b**) Photograph of the testing environment and equipment. (**c**) Key test parameters for static human pose evaluation, including various pose types, distances, and orientations, all performed using a mannequin. (**d**) Various motion types used to evaluate motion capture performance under dynamic conditions, performed by human subjects.

In practical tests, the coordinate systems of different devices need to be aligned to facilitate the analysis of motion capture data. As illustrated in Figure 1a, the origin of the default coordinate system for Kinect is defined at the center point of its depth camera [38], with the depth direction as the positive *z*-axis, the positive *x*-axis pointing to the right, and the positive *y*-axis pointing downward. The origin of the data coordinate system for the Visualeyez device is located at the center of its central capture lens [35], with the depth direction as the positive *z*-axis, the positive *x*-axis pointing upward, and the positive *y*-axis pointing to the right. During the testing process, the z-axes of both devices were positioned in the same vertical plane and maintained parallel to each other, and the YOZ plane of the Kinect devices and the XOZ plane of Visualeyez were made coplanar.

In the experiments conducted in this study, both Kinect and Visualeyez were used to simultaneously measure the subjects. For the acquired data, they were first transformed into a unified coordinate system. Subsequently, the data acquired from Visualeyez were used as a benchmark to compare and analyze the data obtained from Kinect.

### 2.2. Experimental Scenarios

To comprehensively evaluate the accuracy of human pose data identified by the Kinect, we categorized the testing tasks into static and dynamic scenarios from an application perspective. In the static tests, a mannequin with a height of 1.85 m was selected as the sole subject. This mannequin closely approximates the full-body morphology of a human and can maintain a static posture, as visually compared to a real human in Figure 1b. Consequently, static tests enable the repeatable and precise reproduction of the human pose to be measured, facilitating the direct assessment of discrepancies between motion capture data and ground truth data. This setup was also used to investigate how factors such as the subject’s position, orientation, and posture affect the motion capture accuracy and stability. In the dynamic tests, human subjects performed a variety of pre-defined actions in sequence to evaluate the overall accuracy of the Kinect in continuous motion capture.

#### 2.2.1. Static Test Protocol

The specific tasks and scenarios for the static tests are illustrated in Figure 1c. In these tests, the mannequin was configured in a stable, upright standing posture, further categorized into three distinct poses based on upper limb configuration: standing at attention, arms swinging anteroposteriorly, and arms outstretched laterally. These poses were selected to represent common and typical human postures.

The relative positioning between the subject and the Kinect was limited to the most prevalent application scenario: the subject positioned directly in front of the Kinect. Considering the recognition range of Kinect, the subject was maintained within a distance range of 1500 mm to 4000 mm directly in front of the device. Distances less than 1500 mm could result in the hands exceeding the effective recognition range. Within this range, static tests with the subject directly facing the Kinect were conducted at 100 mm intervals. For tests where the subject was not directly facing the Kinect, intervals of 500 mm were used. In these instances, although the subject’s position remained directly in front of the Kinect’s lens, their body orientation maintained a fixed angular deviation from the Kinect’s negative *z*-axis.

For scenarios where the subject’s orientation deviated from the Kinect’s negative *z*-axis, specific yaw angles of ±15°, ±30°, and ±45° were implemented.

The aforementioned subject-to-device distance refers specifically to the separation along the device’s *Z*-axis. The device’s position is defined as the origin of its coordinate system. The subject’s position is defined by the furthest protrusion of their toes relative to a circle centered at their body’s center. Therefore, when the subject stands directly in front of the device, facing it, the *Z*-axis distance between the toes and the device constitutes the measured distance. By rotating the subject in place, the facing direction can be adjusted while maintaining this distance. This distance was measured using a portable laser rangefinder during the experiments.

Taking into account the aforementioned factors, the specific tasks for the static tests are detailed in Table 1.

#### 2.2.2. Dynamic Test Protocol

The specific tasks and scenarios for the dynamic tests are illustrated in Figure 1d. In these experiments, multiple human subjects, as opposed to the mannequin, participated directly to perform pre-defined actions. We did not investigate scenarios involving multiple subjects simultaneously; rather, similar to the static tests, each experiment involved only one subject present in the scene.

The pre-defined actions comprised four types: marching in place, forward and backward walking, lateral walking, and in-place body rotation. During walking, neither the speed of body movement nor arm movements were strictly controlled (though natural swinging was encouraged); subjects were instructed to act according to their personal habits, provided the task duration was satisfied. Each type of movement only had specific goals related to body movement. For example, forward and backward walking required the subject to begin at a distance of 3500 mm from Kinect, proceed forward to 1500 mm, and then retreat backward to 3500 mm. Marching in place and lateral walking tests were conducted at a series of pre-defined initial positions, with a *z*-axis interval of 500 mm between these positions. The specific tasks for the dynamic tests are detailed in Table 2.

The diversification of test movements was designed according to the principle of including common types of motion while maintaining the complexity of the movements within certain limits. This facilitates the comparison and evaluation of the Kinect’s performance in capturing dynamic motion.

### 2.3. Description and Visualization of Human Pose Data

#### 2.3.1. Selection of Joint Nodes

**Figure 2:**
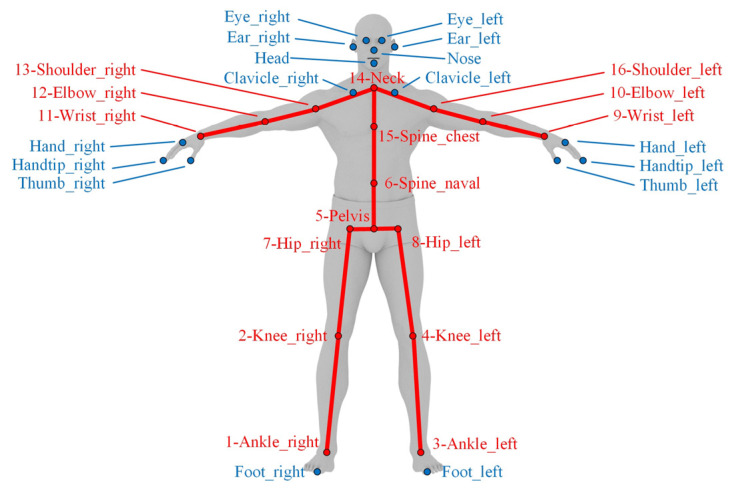
Selection from the 32 human tracking joint nodes provided by Azure Kinect DK, with 16 nodes used to reconstruct the human skeletal morphology. For each selected node, the physical marker required for Visualeyez recognition was attached to the corresponding location on the body surface. Considering that this device necessitates grouping every four markers and connecting them to the receiver via a single cable, adjacent numbering was adopted to minimize the constraints imposed by cables on the subject’s movement.

The Azure Kinect DK device inherently represents the human skeleton using 32 predefined joint nodes. From these, we selected 16 nodes located on the torso and limbs for pose estimation, aiming to retain the maximum amount of human posture data while discarding unnecessary test data. For instance, nodes located on facial features and hands were excluded from our evaluation. Similarly, the clavicle node was omitted due to its proximity to the neck node. The toe node was also excluded due to its demonstrably low recognition accuracy. The final selection of 16 nodes and their corresponding experimental numbering is illustrated in Figure 2.

#### 2.3.2. Ensuring Data Quality of Skeletal Nodes

The spatial positions of skeletal nodes serve as the foundational data for all our analyses. To ensure data quality, a multitude of detailed measures were implemented during the experiments, including:

- Stable testing conditions: The experiments were conducted in an enclosed indoor space with minimal natural light, primarily illuminated by indoor light sources. These sources consisted of LED lights uniformly distributed across the ceiling. The walls were painted white, the floor was wood-textured, and the primary testing area featured adhesive floor markers. The lighting remained constant throughout all experiments. This setup ensured consistent diffuse lighting conditions, similar to typical well-lit indoor environments.
- Specific design of experimental apparatus: In the experimental area, a thin, graduated carpet was fixed to the floor in front of Kinect to visually indicate the distance between any given position and the Kinect. The mannequin used in static experiments was mounted on a mobile stand equipped with counterweights, allowing for convenient and precise adjustments to its position and orientation, thereby enabling corresponding adjustments to the mannequin subject. Curtains were also employed to occlude external light sources that could potentially compromise the measurement accuracy of Visualeyez.
- Experimental data extraction: The data acquisition frequency of the Kinect was set to 30 Hz, while that of the Visualeyez was set to 60 Hz. In static test tasks, the Kinect continuously acquired 500 frames of valid data, which were directly averaged. The Visualeyez, on the other hand, continuously acquired over 1000 frames of data, from which, after preliminary screening and filtering, 100 high-quality frames were selected for averaging. In dynamic test tasks, if the Visualeyez software momentarily failed to effectively detect a visual marker due to occlusion or other factors, the last valid data point corresponding to that marker was used as the current data record. This approach aimed to maximize the integrity of data during dynamic testing. Through this processing, the data for each skeletal node obtained from both devices were refined to minimize the influence of environmental disturbances, such as random infrared interference and occlusion.
- Synchronization of experimental data: Compared to static tests, data acquired from the two devices in dynamic tests required further temporal synchronization. To ensure low-latency data processing, the software systems of the two testing devices were operated on separate computer systems, both of which exhibited startup delays. This presented challenges for strict hardware-based synchronization. Consequently, an offline data synchronization method based on timestamps and pose comparison was adopted. Specifically, we first selected the most recent 500 frames of valid data acquired by the Kinect and designated the first frame as the Kinect’s starting frame. Then, based on the timestamp, the Visualeyez data frame closest in time to the Kinect’s starting frame was identified. Next, within a range of 60 frames before and after this identified Visualeyez frame, the frame exhibiting the closest human pose to the starting data was selected and designated as the Visualeyez starting frame. Through this process, the starting points of the two data sequences were aligned. Subsequently, all data frames were matched based on their respective actual time intervals. The method used to assess the similarity of human posture will be described in the following section.

#### 2.3.3. Visualization of the Human Skeleton

Based on the three-dimensional coordinates of the 16 selected joint nodes and their parent–child relationships, a three-dimensional model of the human skeleton can be rendered for explicit analysis. It is important to note that, given the selected set of joint nodes, the extremities in rendered human model correspond to wrist, ankle, and neck positions.

### 2.4. Motion Capture Data Correction

The spatial positions of joint nodes acquired by Visualeyez were treated as the ground truth data. The discrepancies between these ground truth data and those acquired by Kinect were defined as deviations. This section introduces a correction method for the Kinect data, predicated on the availability of ground truth data. It is essential to emphasize that this study does not delve into optimal correction methodologies or data prediction techniques. Rather, it aims to investigate the potential for post-processing the correction of the inherent deviations in Kinect data.

The correction method is detailed as follows: After transforming the Kinect data into the Visualeyez coordinate system using Equation (1), the transformed data are treated as the independent variables, and the ground truth data as the dependent variables. Fitting is performed separately for each corresponding coordinate axis component. The multivariate linear regression function in Origin 2022 software was employed to perform the fitting calculations and obtain the coefficient and intercept parameters.

(1) $$\left\{\begin{array}{l} {\tilde{x}}_{ki}=-y_{ki}-∆x\\ {\tilde{y}}_{ki}=x_{ki}-∆y\\ {\tilde{z}}_{ki}=z_{ki}-∆z \end{array}\;\right.$$

In Equation (1), $\left\{x_{ki},y_{ki},z_{ki}\right\}$ represents the raw data in the Kinect coordinate system, $\left\{{\tilde{x}}_{ki},{\tilde{y}}_{ki},{\tilde{z}}_{ki}\right\}$ denotes their transformed values in the Visualeyez coordinate system, the subscript i is the joint node index, and $\left\{∆x,∆y,∆z\right\}$ are the offset values, which are obtained using Equation (2),

(2) $$\left\{\begin{array}{l} ∆x=-y_{k5}-x_{v5}\\ ∆y=x_{k5}-y_{v5}\\ ∆z=z_{k5}-z_{v5} \end{array}\;\right.$$

where $\left\{x_{vi},y_{vi},z_{vi}\right\}$ represents the raw data acquired by the Visualeyez, i.e., the ground truth data. The index i=5 signifies that the data of the pelvis joint node were used for calculating the offset values. This transformation ensures that $\left\{{\tilde{x}}_{k5},{\tilde{y}}_{k5},{\tilde{z}}_{k5}\right\}$ and $\left\{x_{v5},y_{v5},z_{v5}\right\}$ have identical values, meaning that the corrected position of the pelvis node coincides with the reference position.

Multivariate linear regression treats each joint node as an independent model. Based on the calculated coefficients $\left\{a_{xi},a_{yi},a_{zi}\right\}$ and the intercept parameters $\left\{b_{xi},b_{yi},b_{zi}\right\}$, the relationship between the corrected data $\left\{x_{fi},y_{fi},z_{fi}\right\}$ and the data $\left\{{\tilde{x}}_{ki},{\tilde{y}}_{ki},{\tilde{z}}_{ki}\right\}$ is further established, as shown in Equation (3).

(3) $$\left\{\begin{array}{l} x_{fi}=a_{xi}*{\tilde{x}}_{ki}+b_{xi}\\ y_{fi}=a_{yi}*{\tilde{y}}_{ki}+b_{yi}\\ z_{fi}=a_{zi}*{\tilde{z}}_{ki}+b_{zi} \end{array}\;\right.$$

### 2.5. Evaluation Method for Motion Capture Data

For a consumer-grade motion capture system such as Kinect, directly evaluating the spatial positional accuracy of skeletal nodes does not have obvious reference significance. On the other hand, the spatial orientation of individual skeletal segments aligns more closely with human intuitive perception, and joint angles between adjacent segments are frequently employed in motion tracking tasks for humanoid characters [39,40]. Therefore, this paper utilizes the similarity of spatial orientations of skeletal segments to quantify the similarity of skeletal images and evaluate the accuracy of motion capture data. Specifically, cosine similarity is adopted as the metric to measure the discrepancy between two vectors. A cosine value approaching 1 indicates a corresponding angle approaching 0°, signifying a higher degree of similarity in the spatial orientation of the two vectors. Furthermore, we introduce weighting factors for skeletal segment cosine similarities to reflect the varying contributions of individual segment similarities to the overall skeletal similarity.

First, the matrices $\left\{{\tilde{X}}_{k},{\tilde{Y}}_{k},{\tilde{Z}}_{k}\right\}$ and $\left\{X_{v},Y_{v},Z_{v}\right\}$, previously arranged according to joint index order, are transformed into matrices $M_{k}$ and $M_{v}$, and arranged according to skeletal segment order using Equation (4). Note that $\left\{{\tilde{X}}_{v},{\tilde{Y}}_{v},{\tilde{Z}}_{v}\right\}$ is equivalent to $\left\{X_{v},Y_{v},Z_{v}\right\}$. Subsequently, the cosine similarity $COS_{j}$ for each skeletal segment and the overall skeletal cosine similarity $COS_{H}$ are obtained using Equations (5) and (6), respectively, where the subscript j denotes the skeletal segment index. The average cosine similarity of the upper limb segments (forearm, upper arm, and shoulder) $COS_{HU}$ and the average cosine similarity of the lower limb segments (shank, thigh, and hip) are calculated analogously to $COS_{H}$. Similarly, based on the corrected values $\left\{X_{f},Y_{f},Z_{f}\right\}$, the corrected cosine similarity for each segment $COS_{fj}$ and the corrected overall skeletal cosine similarity $COS_{fH}$ can be obtained.

(4) $$M_{\left\{k,v\right\}}=\left[\begin{array}{ccc} {\tilde{x}}_{\left\{k,v\right\}1} & {\tilde{y}}_{\left\{k,v\right\}1} & {\tilde{z}}_{\left\{k,v\right\}1}\\ {\tilde{x}}_{\left\{k,v\right\}2} & {\tilde{y}}_{\left\{k,v\right\}2} & {\tilde{z}}_{\left\{k,v\right\}2}\\ \vdots & \vdots & \vdots \\ {\tilde{x}}_{\left\{k,v\right\}16} & {\tilde{y}}_{\left\{k,v\right\}16} & {\tilde{z}}_{\left\{k,v\right\}16} \end{array}\right]-\left[\begin{array}{ccc} {\tilde{x}}_{\left\{k,v\right\}2} & {\tilde{y}}_{\left\{k,v\right\}2} & {\tilde{z}}_{\left\{k,v\right\}2}\\ {\tilde{x}}_{\left\{k,v\right\}7} & {\tilde{y}}_{\left\{k,v\right\}7} & {\tilde{z}}_{\left\{k,v\right\}7}\\ {\tilde{x}}_{\left\{k,v\right\}4} & {\tilde{y}}_{\left\{k,v\right\}4} & {\tilde{z}}_{\left\{k,v\right\}4}\\ {\tilde{x}}_{\left\{k,v\right\}8} & {\tilde{y}}_{\left\{k,v\right\}8} & {\tilde{z}}_{\left\{k,v\right\}8}\\ {\tilde{x}}_{\left\{k,v\right\}5} & {\tilde{y}}_{\left\{k,v\right\}5} & {\tilde{z}}_{\left\{k,v\right\}5}\\ {\tilde{x}}_{\left\{k,v\right\}5} & {\tilde{y}}_{\left\{k,v\right\}5} & {\tilde{z}}_{\left\{k,v\right\}5}\\ {\tilde{x}}_{\left\{k,v\right\}5} & {\tilde{y}}_{\left\{k,v\right\}5} & {\tilde{z}}_{\left\{k,v\right\}5}\\ {\tilde{x}}_{\left\{k,v\right\}5} & {\tilde{y}}_{\left\{k,v\right\}5} & {\tilde{z}}_{\left\{k,v\right\}5}\\ {\tilde{x}}_{\left\{k,v\right\}10} & {\tilde{y}}_{\left\{k,v\right\}10} & {\tilde{z}}_{\left\{k,v\right\}10}\\ {\tilde{x}}_{\left\{k,v\right\}16} & {\tilde{y}}_{\left\{k,v\right\}16} & {\tilde{z}}_{\left\{k,v\right\}16}\\ {\tilde{x}}_{\left\{k,v\right\}12} & {\tilde{y}}_{\left\{k,v\right\}12} & {\tilde{z}}_{\left\{k,v\right\}12}\\ {\tilde{x}}_{\left\{k,v\right\}13} & {\tilde{y}}_{\left\{k,v\right\}13} & {\tilde{z}}_{\left\{k,v\right\}13}\\ {\tilde{x}}_{\left\{k,v\right\}14} & {\tilde{y}}_{\left\{k,v\right\}14} & {\tilde{z}}_{\left\{k,v\right\}14}\\ {\tilde{x}}_{\left\{k,v\right\}15} & {\tilde{y}}_{\left\{k,v\right\}15} & {\tilde{z}}_{\left\{k,v\right\}15}\\ {\tilde{x}}_{\left\{k,v\right\}6} & {\tilde{y}}_{\left\{k,v\right\}6} & {\tilde{z}}_{\left\{k,v\right\}6}\\ {\tilde{x}}_{\left\{k,v\right\}14} & {\tilde{y}}_{\left\{k,v\right\}14} & {\tilde{z}}_{\left\{k,v\right\}14} \end{array}\right]$$

(5) $$COS_{j}=\frac{M_{kj}*M_{vj}}{\left\|M_{kj}\right\|\times \left\|M_{vj}\right\|}$$

(6) $$COS_{H}=\frac{\sum_{j=1}^{16}\left(COS_{j}*W_{j}\right)}{\sum_{j=1}^{16}W_{j}}$$

where $W_{j}$ represents the weight matrix corresponding to each skeletal segment, with its values detailed in Table 3. Considering that the Kinect exhibits relatively higher recognition accuracy for the torso region and larger deviations at the extremities, such as hands and feet, the weights of the terminal skeletal segments are set to the highest values. This weighting scheme aims to reflect the contribution of each skeletal segment to the overall recognition accuracy and to more prominently demonstrate the recognition and correction status of the limbs.

## 3. Results

### 3.1. Results of Static Tests

#### 3.1.1. Cosine Similarity Data

**Figure 3:**
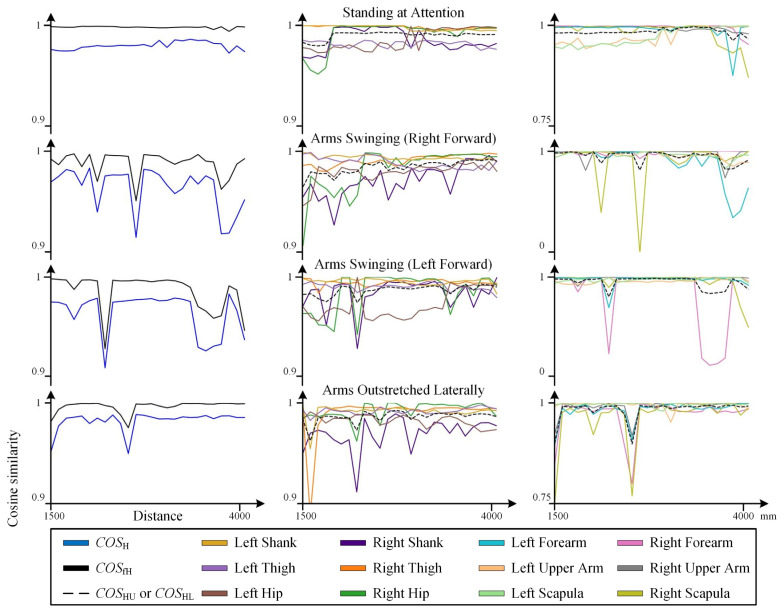
Variation in cosine similarity with distance in static tests when the subject faced the Kinect. The figure includes $COS_{j}$, $COS_{H}$, $COS_{fH}$, $COS_{HU}$, and $COS_{HL}$ data. $COS_{j}$ data are separately visualized for the upper and lower limbs to correspond to the distinct data variation ranges.

**Figure 4:**
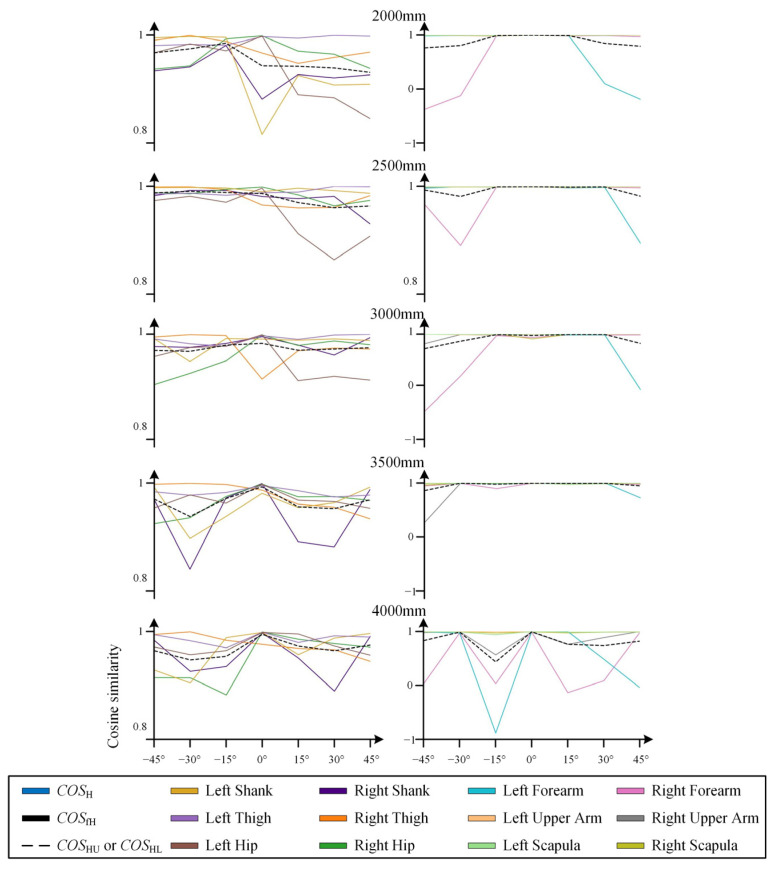
Variation in cosine similarity with orientation in static tests when the subject maintained an arms-outstretched pose at a fixed position. The figure includes $COS_{j}$, $COS_{H}$, $COS_{fH}$, $COS_{HU}$, and $COS_{HL}$ data. $COS_{j}$ data are separately visualized for the upper and lower limbs to correspond to the distinct data variation ranges.

The cosine similarity data obtained from static tests are presented in Figure 3 and Figure 4, with the raw data provided in Appendix A Table A1, Table A2, Table A3 and Table A4.

#### 3.1.2. Visualized Skeletal Data

(1) Distance variation

**Figure 5:**
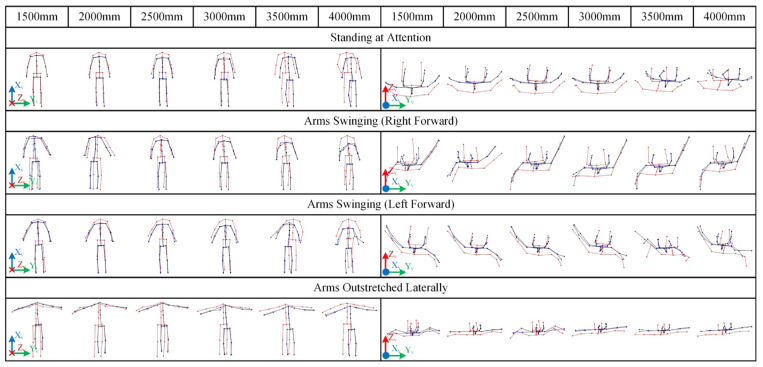
Visualization of skeletal data corresponding to various distances when the subject maintained pre-defined poses and faced the Kinect directly. The left panel shows the frontal view, and the right panel shows the top view. Blue lines correspond to the ground truth data $\left\{X_{v},Y_{v},Z_{v}\right\}$ obtained from the Visualeyez, red lines correspond to the data $\left\{{\tilde{X}}_{k},{\tilde{Y}}_{k},{\tilde{Z}}_{k}\right\}$ obtained from the Kinect, and black lines correspond to the corrected values $\left\{X_{f},Y_{f},Z_{f}\right\}$.

The visualized skeletal data obtained from static tests when the subject faced the Kinect are shown in Figure 5, with the raw data provided in Appendix A Table A1.

(2) Orientation variation

**Figure 6:**
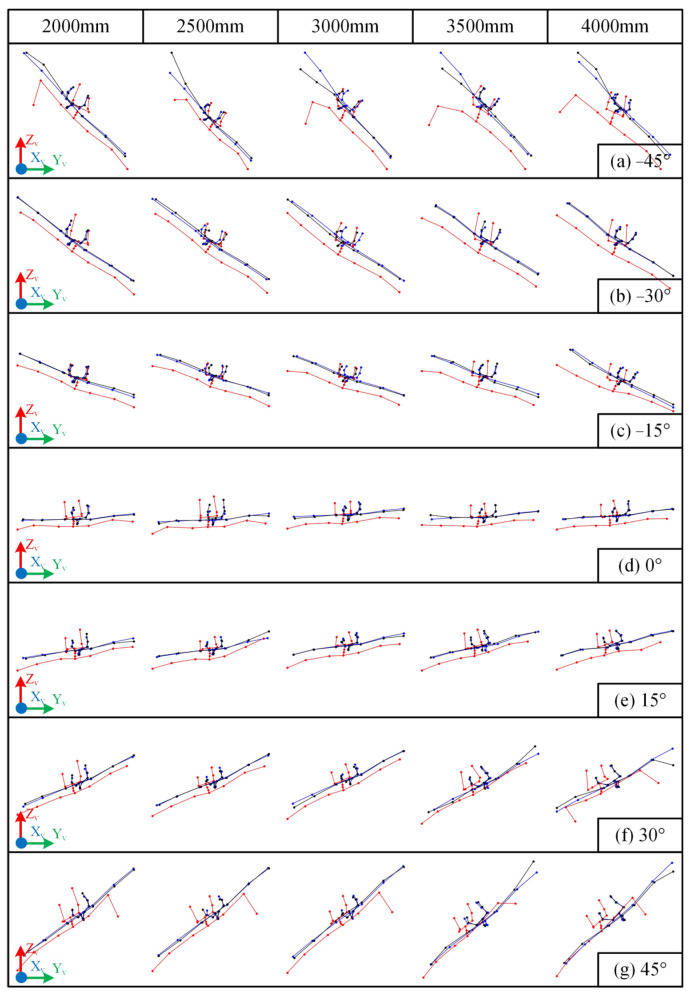
Visualization of skeletal data from a top-down perspective when the subject maintained an arms-outstretched pose while varying their orientation and position. From top to bottom, the subject’s orientation changes from −45° to 45°. The line colors have the same meaning as in Figure 5: blue lines correspond to the ground truth data $\left\{X_{v},Y_{v},Z_{v}\right\}$, red lines correspond to the raw values $\left\{{\tilde{X}}_{k},{\tilde{Y}}_{k},{\tilde{Z}}_{k}\right\}$, and black lines correspond to the corrected values $\left\{X_{f},Y_{f},Z_{f}\right\}$.

The visualized skeletal data obtained from static tests when the subject maintained an arms-outstretched pose and a specific orientation are shown in Figure 6. The raw data for the 2000 mm distance are provided in Appendix A Table A2.

### 3.2. Analysis of Static Test Results

#### 3.2.1. Data Accuracy Analysis

(1) Influence of Distance

As shown in Figure 5, the recognized values $\left\{{\tilde{X}}_{k},{\tilde{Y}}_{k},{\tilde{Z}}_{k}\right\}$ from the Kinect exhibit three-dimensional spatial deviations from their ground truth $\left\{X_{v},Y_{v},Z_{v}\right\}$, with relatively larger deviations observed along the *x*-axis. However, for a variety of static poses, the skeletal morphology directly obtained by the Kinect (red) generally resembles the ground truth skeletal morphology (blue). Combined with the cosine similarity results in Figure 3, it can be inferred that within the distance range of 2000 mm to 3500 mm, the Kinect demonstrates good recognition accuracy. Both excessively close and excessively distant positions may lead to a decline in the accuracy of the Kinect’s skeletal recognition.

For example, in the test involving the arms swinging (left arm forward) pose, when the distance was less than 3300 mm, $COS_{H}$ remained at an excellent level of approximately 0.97. However, when the distance exceeded 3300 mm, it decreased to approximately 0.94. In the experiments with the arms-outstretched pose, $COS_{H}$ gradually increased from 0.95 at 1500 mm to 0.98 at 2000 mm and then stabilized around 0.98. This suggests that both excessively close and excessively distant positions relative to the Kinect camera can potentially reduce recognition accuracy.

(2) Influence of Orientation

The cosine similarity results in Figure 4 demonstrate that changes in orientation directly influence the $COS_{H}$ values. For instance, the curves of $COS_{9}$ (Left Forearm) and $COS_{11}$ (Right Forearm) exhibit a pattern of being higher in the middle (when the yaw angle is near 0°) and lower at the extremes (when the yaw angle deviates further from 0°). This indicates that as the yaw angle increasingly deviates from 0°, the accuracy of skeletal morphology recognition decreases, which is consistent with the skeletal morphology recognition results shown in Figure 6.

Figure 6 also clearly shows that within the range of ±[0°~15°], particularly at 0°, the recognition of the human skeleton is good. Within the range of ±[15°~30°], the recognition of most joints is good. However, as the distance increases, some joints exhibit larger recognition errors. For example, at a distance of 4000 mm and an orientation of 30°, the position of one of the wrists exhibited a significant unexpected deviation. As the yaw angle further increases, this phenomenon becomes more prevalent. For instance, at an orientation of ±45°, significant deviations in the position of one of the wrists appear across all distances. This significant deviation in joint position recognition can also affect the representation of skeletal joint angles. For example, at an orientation of +45°, the Kinect’s recognition of the spatial position of the left wrist node showed a large error, which also led to a deviation in the left elbow joint angle.

This phenomenon of significant recognition deviations at large orientation angles occurs more frequently at distances exceeding 3000 mm. However, even within the 2000 mm to 3000 mm range, which is empirically considered to generally provide good recognition, a small number of experimental groups exhibited this issue.

(3) Influence of Skeletal Distribution

The $COS_{j}$ data reveal that the Kinect demonstrates good recognition accuracy for the human torso, while larger errors and a corresponding decrease in accuracy are more likely to occur in the limbs. Notably, the recognition accuracy of the Kinect for the upper limbs is significantly lower than that for the lower limbs, as clearly evidenced by the results presented in Figure 3 and Figure 4. For example, in the first two tests in Figure 3, $COS_{11}$ (Right Forearm) and $COS_{12}$ (Right Upper Arm), and in the latter two tests, $COS_{9}$ (Left Forearm) and $COS_{10}$ (Left Upper Arm), each exhibited poor performance and significantly impacted the overall cosine similarity.

**Figure 7:**
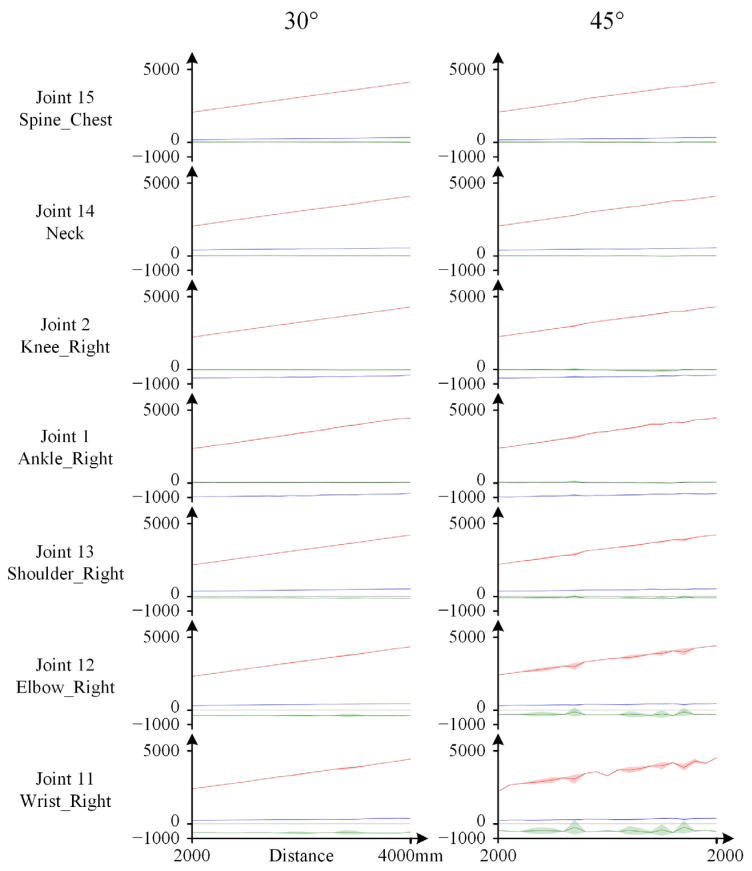
Values and standard deviations of $\left\{{\tilde{X}}_{k},{\tilde{Y}}_{k},{\tilde{Z}}_{k}\right\}$ when the subject maintained an arms-outstretched pose. The left panel shows the results for an orientation deviation of 30°, and the right panel shows the results for an orientation deviation of 45°. The values of ${\tilde{x}}_{ki}$, ${\tilde{y}}_{ki}$, and ${\tilde{z}}_{ki}$ are represented by blue, green, and red lines, respectively, and their standard deviations are represented by shaded areas of the corresponding colors.

The standard deviation data corresponding to the different joint values $\left\{{\tilde{X}}_{k},{\tilde{Y}}_{k},{\tilde{Z}}_{k}\right\}$ reveal the direct cause of the deviations that tend to occur at the terminal joints of the limbs. Figure 7 sequentially presents the standard deviation data for the spatial distances of two torso joint nodes (Spine_Chest, Neck), two lower limb joint nodes (Knee_Right, Ankle_Right), and three upper limb joint nodes on the same side as the lower limbs (Shoulder_Right, Elbow_Right, and Wrist_Right). In the results shown in Figure 7 for a yaw angle of 30°, the standard deviations of all data are very small. However, as the yaw angle increases to 45°, the fluctuation in the standard deviation of the spatial position data of limb joints, especially the terminal joints, increases significantly, leading to a corresponding decrease in the recognition accuracy of these joints. The significant bending of the terminal bones of the arm in the skeletal morphology shown in Figure 6 can be attributed to this phenomenon. Furthermore, this high standard deviation also implies that instantaneous results for the terminal joints of the limbs should not be overly relied upon when the yaw angle is large, even if they appear to be relatively accurate at that moment.

Moreover, the joint position standard deviation data shown in Figure 7 further confirm that the Kinect’s recognition accuracy for the torso remains consistently high. This is because, under various distance and orientation configurations, the standard deviation of the position data of torso joints remains consistently low.

#### 3.2.2. Effectiveness of Data Correction

The effectiveness of the correction applied to the Kinect data is clearly observable from the graphical representation of the skeletal data. In Figure 5, the corrected human skeletal data, represented by black lines, although still retaining the characteristic bending at the arms, are significantly closer to the ground truth morphology represented by blue lines than the original morphology represented by red lines. Moreover, the magnitude of this unexpected bending is also markedly reduced. Additionally, the forward-leaning deviation present in the original data is mitigated to a certain extent. Similar results are observed in Figure 6, where the initially pronounced arm deviations are substantially suppressed. Therefore, the Kinect recognition deviations, influenced by multiple factors such as distance and orientation, can be reduced to varying degrees through fitting-based correction.

The changes in cosine similarity before and after correction also corroborate this effect (detailed data are provided in Appendix A Table A3 and Table A4). The $COS_{fH}$ values after fitting correction show an improvement rate ranging from 26% to 74% compared to the $COS_{H}$ values before correction, indicating an effective enhancement in recognition accuracy across all test groups. However, it should be noted that with the multivariate linear regression method employed in this study, certain characteristics of the original data are still partially retained, meaning that deviations cannot be completely eliminated.

### 3.3. Results of Dynamic Tests

**Figure 8:**
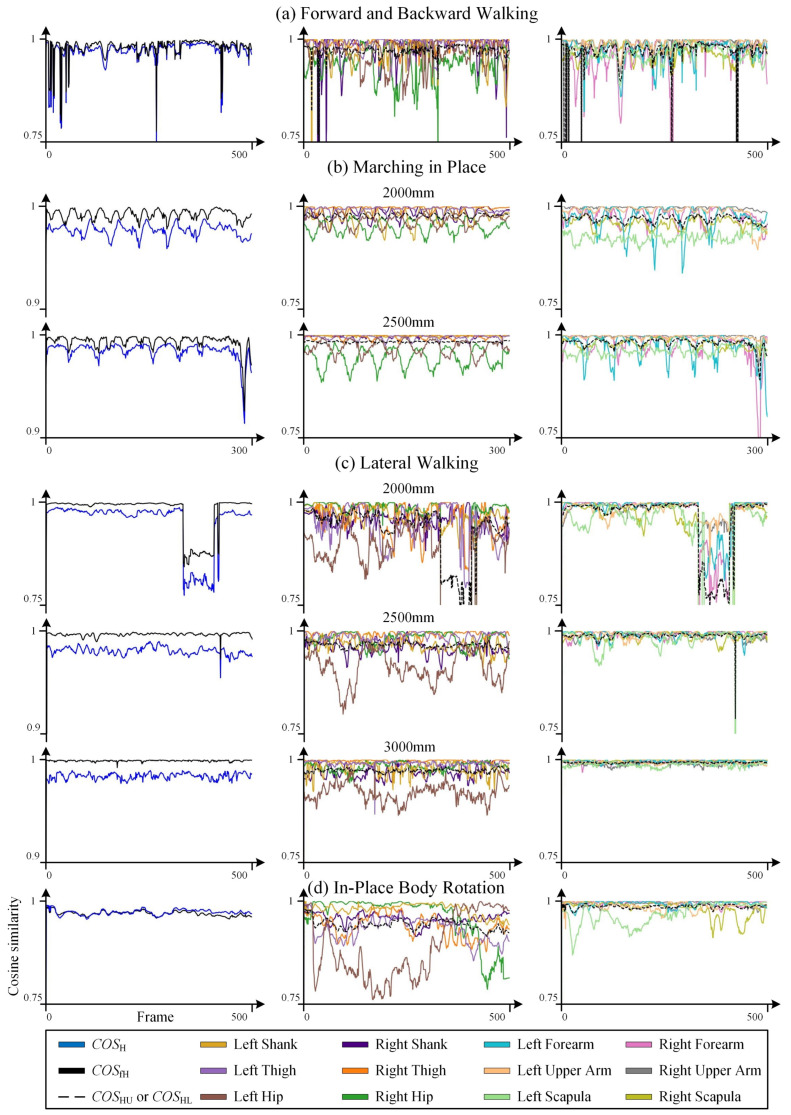
Cosine similarity results of dynamic tests, where (**a**) corresponds to the forward and backward walking test, (**b**) corresponds to the marching in place test, (**c**) corresponds to the lateral walking test, and (**d**) corresponds to the in-place body rotation test. The data frame index corresponding to the data sampling time is used as the horizontal axis, uniformly arranged to facilitate retrieval of data records. In total, 500 frames correspond to a sampling duration of approximately 17 s. The figure includes $COS_{H}$, $COS_{fH}$, and $COS_{j}$ data, with $COS_{j}$ data presented separately for the upper and lower limbs for comparison with static test results.

The cosine similarity results obtained from the various dynamic tests are presented in Figure 8, while the graphical skeletal data are provided in video format in the Appendix A Materials. The marching in place and forward and backward walking tests were performed by a 24-year-old male subject with a height of 1.85 m and a weight of 70 kg. The lateral walking and in-place body rotation tests were performed by another 25-year-old male subject with a height of 1.78 m and a weight of 71 kg. These two human subjects had comparable heights and physiques to the aforementioned static mannequin and wore clothing similar to that of the mannequin, thereby facilitating the correlation and comparative analyses between the dynamic and static test results.

### 3.4. Analysis of Dynamic Tests

(1) Influence of Distance in the Depth Direction

The results of the forward and backward walking test provide the most direct evidence of the correlation between distance in the depth direction and recognition accuracy. In Figure 8a, the data frame index on the horizontal axis corresponds to the complete process of the subject walking forward from a distance of 3500 mm to 1500 mm and then immediately retrogressing to 3500 mm. When the subject was within the intermediate distance range of [2000 mm~3500 mm], the recognition results are stable and good ($COS_{H}>0.9$). However, when the subject was too close or too far, the recognition results exhibit significant fluctuations, and the accuracy decreased ($COS_{H}>0.75$). The trend of recognition accuracy with respect to distance in the depth direction and the stable range is consistent with the results of the static tests, although the accuracy is slightly lower than that of the static standing tests.

The marching in place test further demonstrates the sensitivity of recognition accuracy to distance variations in the depth direction under dynamic conditions. In the results of the marching in place test at a distance of 2500 mm (the first 300 frames) shown in Figure 8b, as the arms swing back and forth, the accuracy variations of the arm on the same side are consistent. For example, the variations in the left forearm and the left upper arm are synchronized, and the accuracy of the forearm is significantly lower than that of the upper arm ($COS_{9}<COS_{10}$), which is consistent with the difference in the range of motion in the depth direction between these two skeletal segments. Moreover, the variation patterns of the two arms alternate. However, it should be noted that when the depth distance is reduced to 2000 mm, the recognition accuracy of the shoulder joint begins to decrease as the shoulder data approaches the upper edge of the image. At this point, the recognition deviation of the shoulder bones (Left Scapula, Right Scapula) becomes significantly higher than that of the upper arm and close to that of the forearm. This is also the reason why the recognition accuracy of the hip bones (Left Hip, Right Hip), which are closer to the center of the image, does not change significantly as the distance changes from 2500 mm to 2000 mm.

(2) Influence of Lateral Distance

The trend of recognition accuracy with respect to lateral distance also reveals the existence of an optimal range of [−0.5 m~0.5 m], corresponding to the position directly in front of the Kinect lens. As the subject moves laterally away from the lens, the recognition accuracy decreases. In the lateral walking experiment shown in Figure 8c, the subject initially faced the Kinect lens. The test began with the subject taking a sidestep to the left boundary, then walking to the right, passing the initial position, and continuing to the right boundary, and finally walking back to the left to the initial position. Taking the test at a z-direction distance of 2500 mm as an example, during this process, the recognition performance in the central region [−0.5 m~0.5 m] was excellent ($COS_{H}>0.95$), while at the two extreme sides, the recognition accuracy significantly decreased ($COS_{H}>0.74$).

The lateral walking test further validates the influence of image edge factors on recognition accuracy. At distances of 2500 mm and 3000 mm, since the ankle joint is closer to the bottom of the image than the knee joint, the recognition accuracy of the lower leg bones is generally lower than that of the upper leg bones (consistent with the results of the static tests), i.e., $COS_{1}<COS_{2}$ and $COS_{3}<COS_{4}$. However, when the distance is further reduced to 2000 mm, as the subject moves laterally, both the ankle and knee joints approach the side edges of the image, causing the recognition deviation of the upper leg bones to become similar to, and in some areas even exceed, that of the lower leg bones, i.e., $COS_{1}\geq COS_{2}$ and $COS_{3}\geq COS_{4}$.

(3) Influence of Occlusion

Occlusion is another important factor that directly affects recognition accuracy, as demonstrated in the in-place body rotation test. When the human body is oriented sideways to the Kinect lens, some body regions are occluded by the body itself, leading to recognition deviations. In the in-place body rotation experiment shown in Figure 8d, the subject initially faced the Kinect lens, then rotated to the left and returned to the initial position, and then rotated to the right and returned to the initial position. The rotation angle was approximately [−15°~15°]. Furthermore, unlike the static tests, the upper limbs remained naturally hanging down during the rotation. During this process, the overall trend of $COS_{H}$ was similar to that of the static tests, with the highest recognition accuracy when facing the Kinect directly ($COS_{H}>0.95$) and the lowest at the maximum rotation angle ($COS_{H}\approx 0.76$). During rotation, the hip and shoulder regions are most susceptible to occlusion due to their minimal corresponding body area and their location on the opposite side of the body. This is directly reflected in the lowest recognition accuracy of the hip and shoulder bones, with the accuracy directly correlating with the rotation angle.

In the aforementioned process, although the subject was positioned directly in front of the Kinect lens and was not near the lateral edges of the image, the phenomenon of the recognition deviation of the upper leg bones exceeding that of the lower leg bones, as observed in the lateral walking test, occurred again. We believe that as the rotation angle increases, not only is the hip joint most affected by occlusion, but partial occlusion of the upper leg region also leads to deviations in the Kinect’s recognition of the knee joint position. These factors directly affect the recognition accuracy of the upper leg bones. During rotation, the variation in recognition accuracy of the upper leg bones showed a certain consistency with that of the hip bones, such as the right hip bone (Right Hip) and the right upper leg bone (Right Thigh), which is consistent with our analysis.

(4) Effectiveness of Correction

Observing $COS_{H}$ and $COS_{fH}$ in Figure 8, it can be seen that after correcting the dynamic test data, the overall cosine similarity results improved by about 2% (calculation data are provided in Appendix A Table A5), indicating a very limited improvement. This is partly because, in the dynamic test results, $COS_{H}$ itself maintained a relatively high level in most cases, leaving limited room for improvement. On the other hand, in regions with poor test results, the data fluctuate more drastically, which significantly limits the effectiveness of the linear fitting correction method employed in this study.

## 4. Discussion

(1) Recognition Accuracy

At the outset of this study, we were intrigued by the question of whether a body in absolute rest or a body in continuous motion corresponds to higher recognition accuracy for the Kinect. By comparing the results of the in-place body rotation test in dynamic tasks (Figure 8d) and the orientation test in static tasks (Figure 4, 2000 mm), it can be observed that the $COS_{HL}$ value for the lower limbs in the static condition remains above 0.95 within the [−45°~45°] range. In contrast, even within the [−15°~15°] range in the dynamic condition, the $COS_{HL}$ partially drops to 0.9. Therefore, it is evident that the Kinect camera exhibits higher recognition accuracy for a human skeleton in a static state. It is important to note that this comparison is based on the $COS_{HL}$ value, representing the comprehensive cosine similarity of the six skeletal segments of the lower limbs, rather than the whole-body cosine similarity $COS_{H}$. This is because the upper limb postures in the two test tasks were not consistent.

Furthermore, regardless of whether the subject’s body is in a static or dynamic state, factors such as the distance and orientation relative to the Kinect camera, as well as self-occlusion, have a direct impact on recognition accuracy. The regions of high $COS_{H}$ values, which were experimentally determined, are recommended for preferential use. Specifically, these include maintaining a distance in the depth direction within the range of [2000 mm~3000 mm], keeping the lateral distance as close as possible to [−0.5 m~0.5 m], and maintaining an orientation directly facing the Kinect or within the range of [−15°~15°]. Under these conditions, the $COS_{H}$ value can typically be maintained above 0.9.

The recognition accuracy of bones located at the extremities of the limbs is generally lower than that of their parent bones. However, under certain circumstances, the opposite may occur, with the recognition accuracy of the parent bones significantly decreasing. These circumstances include the occurrence of visual occlusion (e.g., during rotation), excessively close depth distances (shoulder approaching the upper edge of the image), and excessively large lateral displacement (limbs approaching the side edges of the image).

(2) Limitations of the Tests

It is worth noting that this study did not employ a large number of human subjects, and the tested movements were relatively conventional. This was constrained by the diversity and uncertainty of the issues discussed in this paper. Subjects with varying individual characteristics exhibit personalized manifestations in terms of body morphology, movement behaviors, and preferred movement patterns. This paper primarily focuses on analyzing the impact of active, usage-related factors on the accuracy of Kinect’s built-in motion capture for a given adult user. These factors include relative distance and orientation to the device, occlusion, and typical movement behaviors. Factors associated with passive individual physical characteristics were not examined under a systematic testing and analysis in this study. Consequently, we intentionally selected static mannequins and human subjects with similar physiques and statures, which facilitated the analysis of the aforementioned active factors in the extensive datasets. It is worth noting that the relatively small sample size of subjects may introduce certain limitations to the generalizability of our findings. Nevertheless, we conducted some additional exploratory tests, and preliminary results indicate that the findings and discussions regarding the active factors demonstrate good robustness, provided that height and physique do not undergo substantial variations.

Furthermore, the tests were conducted under essentially constant indoor lighting conditions, primarily illuminated by artificial light sources. While this condition is similar to typical scenarios with ample natural light and aligns with the common usage of the device, it is acknowledged that conclusions drawn from this single condition may have potential limitations. Although we have considerable confidence in the robustness of the aforementioned conclusions regarding accuracy, variations in lighting and scene conditions could potentially influence the overall findings.

In summary, this paper focuses on investigating the overall performance of Azure Kinect DK in terms of motion recognition accuracy. In the future, with more specific and refined test objectives, a larger number and variety of subjects, as well as more diverse movements, will be considered. Specifically, further evaluation is needed regarding Kinect’s performance in capturing more complex or faster movements.

(3) Data Correction

After applying linear regression correction to the spatial position data of skeletal nodes obtained by the Kinect, the $COS_{fH}$ in static tests showed a significant improvement compared to $COS_{H}$, while the improvement in dynamic tests was very limited. This is mainly attributed to two reasons. First, the static tests covered a wider range of distances and orientations, resulting in a more pronounced decrease in recognition accuracy at the respective boundary regions. This provided more potential for accuracy optimization. In contrast, the dynamic tests had a smaller test range, and the recognition accuracy remained relatively high throughout. Second, in dynamic tests, the continuously reciprocating ground truth data limited the effectiveness of the linear fitting method.

Although the data correction method employed in this study demonstrated overall effectiveness, its rapid deployment and application are clearly restricted. This is due to factors such as the need for additional equipment and steps, as well as inter-individual variability in correction parameters, all of which are contrary to the “plug-and-play” and “anytime, anywhere” advantages of the Kinect device. Nevertheless, we believe that this fundamental correction method and its preliminary results still hold value for informing the future development of more intelligent and convenient correction methods. It should be reiterated that the primary purpose of data correction in this study was to assess whether the accuracy of raw data obtained from the Kinect motion capture device can be significantly improved when precise ground truth data are available. The results of this study provide an affirmative answer to this question. Moreover, in future in-depth studies on the dynamic performance of Kinect, advanced methods for improvement will be concurrently considered.

(4) Implications of the Results for Kinect Application Research

The latest Azure Kinect DK device boasts advancements in camera resolution and depth data accuracy compared to its predecessors. However, it remains a consumer-grade, cost-effective motion capture device. When deployed extensively and independently in real-world applications such as medical treatment, rehabilitation, and real-time robot control, its inherent limitations in human skeletal recognition accuracy cannot be overlooked.

The results presented in this paper realistically reflect these limitations. For example, a subject maintaining a walking motion while regressing from 2000 mm to 3500 mm in the depth direction relative to the Kinect can experience an approximately 17% difference in recognition accuracy. This should be taken into account in applications that demand precise motion capture.

To pursue high-precision Kinect motion capture, this paper proposes recommended usage regions based on experimental results, providing actionable guidelines for researchers and developers utilizing this device. For instance, while Microsoft, the manufacturer of the Kinect device, suggests an optimal depth range of [0.5 m~3.86 m], this study further refines this range to [2 m~3.5 m] to acquire the most accurate representation of the complete human skeleton.

Furthermore, in application tasks where high-precision reference values can be obtained, such as in specific large-area human motion capture scenarios involving a high-precision professional motion capture system and multiple Kinect devices, the skeletal data directly acquired by the Azure Kinect DK can be further corrected based on its pre-acquired reference values to achieve higher accuracy. Even if the raw Kinect data are difficult to correct directly, their accuracy performance and potential for improvement can still be assessed.

Finally, it should be noted that the analysis of whole-body cosine similarity in this paper introduced weighting factors for different skeletal segments. However, the specific setting of these weights may depend on the researcher’s specific task and interests. Therefore, the raw data of unweighted joint nodes from multiple tests are also provided for researchers to utilize.

## Figures and Tables

**Table 1:** Parameter settings for static experiments.

Pose	Orientation (°)	Distance Range (mm)	Distance Interval (mm)	Number of Sub-Tests
Standing at attention	0	[1500, 4000]	100	26
	±30	[1500, 4000]	500	12
	±45	[1500, 4000]	500	12
Arms swinging (Left forward)	0	[1500, 4000]	100	26
Arms swinging (Right forward)	0	[1500, 4000]	100	26
Arms Outstretched laterally ^1^	0	[1500, 4000]	100	26
	±15	[2000, 4000]	500	10
	±30	[2000, 4000]	500	10
	±45	[2000, 4000]	500	10

^1^ For tests involving the arms outstretched laterally pose with orientations not directly facing Kinect, the minimum distance was set to 2000 mm to prevent wrists from exceeding the recognition range.

**Table 2:** Parameter settings for dynamic experiments.

Action ^1^	Orientation (°)	Distance Range (mm)	Distance Interval (mm)	Number of Sub-Tests
Marching in place	0	[1500, 3500]	500	5
Forward and backward walking	0	[1500, 3500]	-	3
Lateral walking	0	[1500, 3500]	500	5
In-place body rotation	[−15,15]	2000	-	1

^1^ Each set of experiments was repeated three times for cross-validation and to mitigate potential data anomalies caused by device-related issues.

**Table 3:** Skeletal weights for whole–body cosine similarity computation.

Skeletal Segment Index (*j*)	Skeletal Segment Name	Terminating Node (*i*)	Originating Node (*i*)	Weight (Pelvis Weight = 1)	Normalized Weight ($W_{j}$)
1	Right Shank	Ankle_Right (1)	Knee_Right (2)	10	0.130
2	Right Thigh	Knee_Right (2)	Hip_Right (7)	5	0.065
3	Left Shank	Ankle_Left (3)	Knee_Left (4)	10	0.130
4	Left Thigh	Knee_Left (4)	Hip_Left (8)	5	0.065
5	Pelvis	Pelvis (5)	Pelvis (5)	1	0.013
6	Lumbar Spine	Spine_Naval (6)	Pelvis (5)	1	0.013
7	Right Hip	Hip_Right (7)	Pelvis (5)	3	0.039
8	Left Hip	Hip_Left (8)	Pelvis (5)	3	0.039
9	Left Forearm	Wrist_Left (9)	Elbow_Left (10)	10	0.130
10	Left Upper Arm	Elbow_Left (10)	Shoulder_Left (16)	5	0.065
11	Right Forearm	Wrist_Right (11)	Elbow_Right (12)	10	0.130
12	Right Upper Arm	Elbow_Right (12)	Shoulder_Right (13)	5	0.065
13	Right Scapula	Shoulder_Right (13)	Neck (14)	3	0.039
14	Cervical Spine	Neck (14)	Spine_Chest (15)	2	0.025
15	Thoracic Spine	Spine_Chest (15)	Spine_Naval (6)	1	0.013
16	Left Scapula	Shoulder_Left (16)	Neck (14)	3	0.039

## Data Availability

The original contributions presented in this study are included in the article/Supplementary Materials. Further inquiries can be directed to the corresponding author.

## Supplementary Materials

The following supporting information can be downloaded at: https://www.mdpi.com/article/10.3390/s25041047/s1, Video S1: Accuracy performance of Azure Kinect DK in multiple dynamic tests.

## Appendix A

**Table A1:** Spatial positioning data of body nodes for the mannequin maintaining an arms-outstretched pose and facing the Kinect camera directly in static tests.

Distance (mm)	Node Index (*i*)	Node Name	Kinect Measurements			Visualeyez Measurements			Corrected Kinect Values		
			${\tilde{x}}_{ki}$ (mm)	${\tilde{y}}_{ki}$ (mm)	${\tilde{z}}_{ki}$ (mm)	$x_{vi}$ (mm)	$y_{vi}$ (mm)	$z_{vi}$ (mm)	$x_{fi}$ (mm)	$y_{fi}$ (mm)	$z_{fi}$ (mm)
1500	1	Ankle_Right	−1181.91	−79.75	1565.90	−945.49	9.29	1352.81	−966.19	8.06	1490.07
	2	Knee_Right	−762.39	−76.92	1351.41	−645.44	−14.54	1305.08	−663.76	−20.16	1398.85
	3	Ankle_Left	−1171.43	143.46	1577.87	−947.15	189.16	1400.66	−970.04	194.94	1506.22
	4	Knee_Left	−747.49	154.39	1384.04	−643.39	197.34	1313.86	−660.91	203.10	1393.28
	5	Pelvis	−272.35	17.07	1302.66	−237.66	85.70	1243.17	−248.41	84.80	1274.80
	6	Spine_Naval	−64.36	11.12	1239.58	77.10	55.12	1187.34	70.21	52.67	1209.00
	7	Hip_Right	−278.87	−82.18	1290.54	−235.36	1.53	1238.37	−246.64	−4.78	1273.75
	8	Hip_Left	−265.12	127.14	1316.11	−221.42	159.01	1260.53	−232.98	163.02	1289.47
	9	Wrist_Left	217.50	753.55	1218.26	275.09	759.95	1311.03	263.01	757.06	1297.46
	10	Elbow_Left	276.75	506.28	1341.67	301.80	507.06	1285.75	306.25	513.11	1377.20
	11	Wrist_Right	136.45	−690.55	1082.30	172.24	−632.04	1212.12	180.39	−628.13	1208.42
	12	Elbow_Right	245.52	−507.94	1273.79	249.26	−382.55	1222.50	258.53	−403.27	1326.40
	13	Shoulder_Right	284.20	−189.53	1152.06	357.67	−103.52	1232.79	358.65	−112.37	1233.73
	14	Neck	367.47	5.60	1168.39	408.86	47.26	1227.52	413.65	48.41	1244.67
	15	Spine_Chest	104.77	4.85	1200.00	199.85	49.11	1173.96	197.29	45.50	1190.52
	16	Shoulder_Left	284.85	215.78	1171.49	358.02	215.53	1237.51	358.60	226.91	1243.46
2000	1	Ankle_Right	−1016.73	−125.23	1971.81	−920.77	−5.35	1873.28	−915.52	−14.01	1901.04
	2	Knee_Right	−622.24	−105.71	1795.11	−619.80	−29.72	1825.31	−616.54	−33.76	1842.45
	3	Ankle_Left	−1008.72	56.36	1988.34	−922.10	175.26	1918.26	−917.52	160.04	1927.88
	4	Knee_Left	−614.77	88.59	1823.68	−588.03	182.16	1832.79	−585.62	173.24	1835.99
	5	Pelvis	−171.81	−9.58	1780.61	−211.96	69.96	1762.81	−209.90	68.89	1759.34
	6	Spine_Naval	21.34	−9.79	1729.99	102.54	37.97	1707.32	104.79	39.16	1706.74
	7	Hip_Right	−175.64	−100.41	1766.37	−209.87	−14.81	1759.38	−207.30	−16.10	1755.45
	8	Hip_Left	−167.56	91.15	1796.40	−196.20	143.03	1778.89	−194.73	142.01	1776.99
	9	Wrist_Left	263.87	726.84	1725.54	297.23	742.99	1823.48	288.25	742.88	1812.57
	10	Elbow_Left	321.61	474.87	1748.98	327.25	491.17	1800.85	326.41	494.07	1795.62
	11	Wrist_Right	139.72	−711.43	1628.11	197.53	−648.90	1740.66	181.54	−641.17	1755.08
	12	Elbow_Right	298.69	−508.78	1680.13	274.45	−399.28	1748.29	282.42	−403.92	1744.10
	13	Shoulder_Right	342.82	−197.11	1665.62	382.95	−120.35	1755.42	381.05	−117.76	1754.38
	14	Neck	418.65	−17.57	1666.19	434.28	30.41	1748.57	437.31	32.63	1751.23
	15	Spine_Chest	178.09	−11.68	1699.94	225.62	31.75	1694.56	228.14	34.32	1698.10
	16	Shoulder_Left	339.89	175.64	1672.99	383.64	198.84	1755.97	381.10	199.85	1751.66
2500	1	Ankle_Right	−1060.83	−152.40	2542.82	−896.70	−25.53	2397.84	−919.05	−27.20	2479.15
	2	Knee_Right	−640.00	−142.17	2343.19	−594.88	−49.29	2351.18	−622.53	−50.99	2390.42
	3	Ankle_Left	−1045.13	26.53	2584.71	−896.53	154.42	2448.32	−919.27	148.08	2540.50
	4	Knee_Left	−631.37	61.69	2384.41	−593.07	162.40	2365.70	−621.28	161.03	2400.63
	5	Pelvis	−155.17	−42.60	2326.97	−186.89	50.98	2292.21	−203.52	49.17	2313.24
	6	Spine_Naval	50.90	−43.01	2265.70	128.16	18.96	2237.43	116.72	17.69	2250.44
	7	Hip_Right	−158.93	−140.87	2314.97	−184.96	−33.98	2287.68	−200.93	−41.23	2310.82
	8	Hip_Left	−150.99	66.39	2340.28	−170.90	123.50	2310.15	−188.23	127.56	2329.06
	9	Wrist_Left	331.05	678.31	2242.97	337.60	720.33	2368.43	324.83	717.11	2337.98
	10	Elbow_Left	386.29	441.51	2380.56	353.28	469.49	2340.65	355.47	473.84	2444.42
	11	Wrist_Right	247.05	−747.45	2113.13	219.94	−670.37	2262.48	219.23	−663.67	2240.85
	12	Elbow_Right	350.42	−560.28	2298.51	300.10	−419.90	2273.81	305.66	−443.84	2379.75
	13	Shoulder_Right	397.92	−243.96	2185.47	407.49	−141.17	2285.11	402.10	−151.02	2281.42
	14	Neck	478.36	−51.11	2199.62	460.13	9.30	2281.55	464.91	9.77	2294.06
	15	Spine_Chest	218.20	−44.62	2226.38	251.19	12.35	2226.67	245.02	12.04	2232.58
	16	Shoulder_Left	404.24	159.23	2204.88	409.50	178.08	2291.34	407.40	188.78	2290.65
3000	1	Ankle_Right	−852.08	−175.45	2945.98	−863.94	−50.71	2905.30	−855.01	−38.38	2887.33
	2	Knee_Right	−469.95	−171.32	2800.78	−573.06	−74.61	2859.93	−565.23	−64.76	2847.92
	3	Ankle_Left	−847.49	18.44	2967.13	−874.69	128.28	2958.82	−865.47	144.84	2933.34
	4	Knee_Left	−460.19	24.55	2850.29	−541.29	136.12	2875.86	−532.86	144.18	2869.76
	5	Pelvis	−42.88	−69.37	2806.34	−165.84	25.53	2803.62	−160.51	33.18	2799.22
	6	Spine_Naval	137.46	−70.52	2750.90	147.82	−6.32	2750.15	151.66	−0.08	2742.88
	7	Hip_Right	−47.41	−155.23	2793.38	−163.57	−59.57	2798.66	−158.42	−50.15	2795.13
	8	Hip_Left	−37.86	25.84	2820.70	−150.30	98.17	2822.96	−143.88	103.89	2816.72
	9	Wrist_Left	390.15	630.44	2756.54	359.58	696.72	2878.24	357.00	691.69	2859.47
	10	Elbow_Left	426.71	387.99	2762.76	374.98	444.69	2852.62	373.64	441.40	2837.05
	11	Wrist_Right	313.27	−776.63	2623.61	242.92	−695.69	2775.43	242.49	−681.90	2752.12
	12	Elbow_Right	379.16	−544.15	2681.01	320.50	−445.11	2786.35	318.57	−431.34	2772.94
	13	Shoulder_Right	439.92	−252.98	2695.11	353.53	−165.63	2795.52	418.14	−157.43	2798.10
	14	Neck	511.48	−84.14	2684.57	482.28	−15.80	2793.51	480.22	−12.73	2787.55
	15	Spine_Chest	284.53	−73.24	2717.53	271.50	−12.78	2738.84	272.92	−7.32	2731.24
	16	Shoulder_Left	458.51	102.08	2711.38	430.74	149.87	2805.81	429.59	150.24	2803.92
3500	1	Ankle_Right	−843.76	−188.58	3469.32	−847.25	−48.32	3422.10	−852.46	−44.75	3417.18
	2	Knee_Right	−449.80	−191.14	3328.33	−555.82	−73.82	3375.94	−558.44	−74.12	3375.36
	3	Ankle_Left	−831.07	−13.75	3492.16	−856.42	131.37	3474.51	−860.17	131.94	3472.68
	4	Knee_Left	−437.04	0.81	3365.65	−522.68	136.17	3392.06	−524.96	133.40	3388.72
	5	Pelvis	−11.58	−88.51	3321.31	−148.71	23.03	3320.42	−148.51	21.76	3321.30
	6	Spine_Naval	173.23	−92.70	3265.50	165.49	−10.89	3266.01	166.10	−14.41	3265.16
	7	Hip_Right	−17.71	−176.22	3307.60	−146.40	−62.23	3314.63	−147.10	−63.18	3315.69
	8	Hip_Left	−4.78	8.77	3336.52	−131.41	95.26	3337.56	−130.91	93.92	3340.30
	9	Wrist_Left	462.71	624.60	3279.20	381.98	690.61	3394.92	396.50	688.59	3390.20
	10	Elbow_Left	487.60	374.90	3274.94	395.81	437.69	3370.75	401.00	433.47	3363.19
	11	Wrist_Right	366.77	−829.63	3213.35	255.05	−700.46	3288.55	261.28	−715.02	3342.78
	12	Elbow_Right	420.85	−581.35	3206.38	335.94	−450.80	3306.32	337.30	−460.17	3312.98
	13	Shoulder_Right	487.41	−284.03	3200.72	441.76	−172.73	3314.69	436.29	−179.48	3310.70
	14	Neck	555.48	−110.10	3194.40	499.63	−22.98	3310.59	500.56	−30.42	3306.36
	15	Spine_Chest	323.87	−98.02	3232.18	288.13	−18.27	3255.94	289.48	−24.07	3253.74
	16	Shoulder_Left	505.21	81.12	3221.72	449.92	143.06	3322.03	448.67	136.11	3321.07
4000	1	Ankle_Right	−801.31	−253.34	3978.51	−825.34	−81.38	3935.26	−839.44	−76.18	3932.71
	2	Knee_Right	−406.94	−248.46	3836.56	−532.70	−106.28	3889.84	−543.99	−101.20	3883.49
	3	Ankle_Left	−788.13	−90.61	4027.84	−833.97	95.88	3989.49	−846.31	101.13	4022.95
	4	Knee_Left	−402.93	−46.36	3880.45	−501.10	101.16	3911.69	−513.32	111.99	3907.12
	5	Pelvis	27.92	−141.49	3834.27	−126.30	−9.39	3836.04	−133.38	−9.89	3841.33
	6	Spine_Naval	214.01	−136.95	3781.66	187.21	−42.88	3781.51	182.55	−43.00	3789.03
	7	Hip_Right	25.93	−229.41	3819.62	−123.78	−95.23	3830.22	−130.47	−96.22	3834.03
	8	Hip_Left	30.12	−44.00	3850.51	−109.25	62.14	3856.45	−117.22	63.12	3862.03
	9	Wrist_Left	470.53	591.76	3800.17	405.81	656.28	3925.66	400.75	671.15	3919.20
	10	Elbow_Left	519.99	345.26	3795.94	418.25	402.75	3895.16	415.55	415.50	3898.40
	11	Wrist_Right	341.20	−814.03	3663.16	276.58	−734.50	3795.31	252.30	−705.27	3793.28
	12	Elbow_Right	446.42	−594.31	3700.88	356.54	−484.36	3812.61	348.79	−470.21	3821.30
	13	Shoulder_Right	563.46	−315.18	3711.85	463.59	−206.19	3825.51	465.35	−201.60	3828.89
	14	Neck	598.04	−136.93	3715.04	521.04	−56.24	3825.01	520.23	−48.70	3836.17
	15	Spine_Chest	365.04	−134.83	3748.48	309.80	−50.18	3769.54	306.80	−48.97	3777.93
	16	Shoulder_Left	562.55	53.83	3740.89	481.37	101.56	3838.78	472.11	117.71	3847.18

**Table A2:** Spatial positioning data of body nodes for the mannequin maintaining an arms-outstretched pose at a 2000 mm distance directly in front of the Kinect camera in static tests.

Orientation (°)	Node Index (*i*)	Node Name	Kinect Measurements			Visualeyez Measurements			Corrected Kinect Values		
			${\tilde{x}}_{ki}$ (mm)	${\tilde{y}}_{ki}$ (mm)	${\tilde{z}}_{ki}$ (mm)	$x_{vi}$ (mm)	$y_{vi}$ (mm)	$z_{vi}$ (mm)	$x_{fi}$ (mm)	$y_{fi}$ (mm)	$z_{fi}$ (mm)
−45°	1	Ankle_Right	−1002.88	32.67	1944.30	−912.39	−49.86	1903.94	−913.48	−30.96	1915.92
	2	Knee_Right	−611.03	−31.96	1789.64	−617.12	−87.00	1869.38	−619.78	−80.45	1859.18
	3	Ankle_Left	−1019.26	123.63	1807.77	−933.89	107.49	1814.97	−934.16	114.50	1832.25
	4	Knee_Left	−610.39	112.46	1704.93	−613.17	76.26	1739.73	−609.15	81.33	1740.91
	5	Pelvis	−171.08	29.98	1717.98	−231.22	−35.49	1747.50	−229.53	−32.78	1757.14
	6	Spine_Naval	17.85	4.95	1669.41	93.81	−85.34	1720.11	95.79	−87.21	1724.48
	7	Hip_Right	−171.88	−51.81	1757.03	−227.17	−101.16	1801.83	−226.21	−93.19	1807.13
	8	Hip_Left	−170.19	120.68	1674.68	−219.66	28.57	1707.60	−216.79	30.60	1722.37
	9	Wrist_Left	197.28	500.94	1114.13	239.60	474.76	1266.09	247.25	478.59	1239.42
	10	Elbow_Left	264.65	346.36	1306.31	276.56	286.76	1443.42	284.31	285.30	1430.12
	11	Wrist_Right	244.73	−421.35	1742.77	239.95	−514.38	2250.50	252.38	−489.63	2255.87
	12	Elbow_Right	314.49	−350.70	1981.76	298.73	−341.52	2079.83	328.91	−321.21	2197.32
	13	Shoulder_Right	361.22	−152.56	1747.47	383.80	−143.28	1857.09	386.83	−136.40	1870.65
	14	Neck	409.08	−27.55	1613.72	424.83	−43.35	1739.21	431.27	−45.70	1746.07
	15	Spine_Chest	172.57	−8.29	1644.84	219.83	−93.25	1712.80	219.86	−97.72	1716.92
	16	Shoulder_Left	323.62	106.88	1485.11	370.54	60.56	1632.37	375.35	53.35	1633.43
−30°	1	Ankle_Right	−1027.16	−6.46	2005.28	−912.53	−63.77	1902.22	−912.58	−48.33	1945.99
	2	Knee_Right	−628.36	−48.36	1844.79	−616.33	−97.55	1861.02	−618.37	−87.02	1863.72
	3	Ankle_Left	−1035.24	125.13	1843.72	−932.03	104.59	1837.98	−935.44	117.76	1849.36
	4	Knee_Left	−623.73	113.94	1726.69	−611.69	83.66	1758.95	−613.72	95.90	1760.02
	5	Pelvis	−182.53	21.48	1738.70	−230.55	−29.99	1749.61	−231.45	−16.89	1750.38
	6	Spine_Naval	9.69	−6.01	1692.63	94.73	−79.55	1716.96	94.49	−65.33	1715.91
	7	Hip_Right	−188.09	−65.68	1767.53	−227.40	−103.37	1793.43	−228.21	−89.90	1789.75
	8	Hip_Left	−176.37	118.14	1706.73	−218.23	39.54	1720.44	−219.07	53.82	1726.17
	9	Wrist_Left	223.48	574.36	1211.85	244.81	546.26	1355.94	235.99	569.39	1348.24
	10	Elbow_Left	275.81	383.91	1380.07	280.29	332.42	1500.62	277.64	352.66	1494.91
	11	Wrist_Right	227.67	−531.99	2026.58	239.14	−580.10	2177.82	241.67	−583.75	2174.6
	12	Elbow_Right	314.40	−426.56	1950.92	299.25	−384.63	2025.80	298.35	−377.30	2021.02
	13	Shoulder_Right	356.32	−182.46	1756.34	384.42	−159.90	1843.82	386.61	−147.36	1842.95
	14	Neck	406.71	−39.92	1638.10	425.76	−43.83	1743.00	427.29	−28.59	1743.18
	15	Spine_Chest	166.07	−24.46	1666.99	221.24	−85.02	1707.89	221.79	−72.08	1706.12
	16	Shoulder_Left	327.91	118.67	1530.04	372.93	79.53	1652.55	374.77	98.46	1651.75
−15°	1	Ankle_Right	−942.66	−81.31	1901.17	−912.67	−81.43	1889.45	−896.32	−62.12	1890.94
	2	Knee_Right	−555.20	−95.50	1787.27	−616.96	−106.27	1843.09	−606.35	−90.28	1829.14
	3	Ankle_Left	−934.45	115.29	1857.14	−930.04	98.13	1874.26	−916.18	119.70	1878.45
	4	Knee_Left	−551.72	93.39	1743.29	−609.89	95.19	1794.02	−598.06	111.90	1794.09
	5	Pelvis	−136.24	9.08	1744.51	−229.85	−15.51	1754.82	−222.57	3.19	1751.84
	6	Spine_Naval	43.26	−5.66	1696.18	94.88	−58.69	1711.41	100.99	−38.06	1707.85
	7	Hip_Right	−138.47	−75.06	1761.87	−227.96	−97.95	1777.76	−220.05	−78.65	1771.85
	8	Hip_Left	−133.76	102.39	1725.26	−216.37	59.28	1745.58	−209.05	78.55	1746.32
	9	Wrist_Left	281.34	640.75	1414.72	255.48	637.33	1528.04	275.03	651.59	1582.82
	10	Elbow_Left	311.36	418.12	1505.85	287.28	393.16	1611.74	291.95	413.50	1628.86
	11	Wrist_Right	284.02	−687.56	1889.89	229.53	−662.53	2023.94	235.90	−657.39	2019.26
	12	Elbow_Right	318.46	−454.31	1821.72	294.19	−435.84	1933.16	277.73	−427.49	1937.79
	13	Shoulder_Right	348.34	−184.93	1707.01	383.02	−172.12	1815.31	385.52	−154.98	1804.95
	14	Neck	410.80	−36.42	1623.69	426.52	−34.47	1748.00	430.28	−15.48	1748.50
	15	Spine_Chest	188.19	−16.37	1662.91	221.63	−62.53	1702.20	226.70	−43.22	1697.91
	16	Shoulder_Left	343.78	139.67	1575.41	375.68	108.79	1691.75	379.76	128.73	1701.92
0°	1	Ankle_Right	−1016.73	−125.23	1971.81	−910.77	−5.35	1873.28	−905.52	−14.01	1901.04
	2	Knee_Right	−622.24	−105.71	1795.11	−619.80	−29.72	1825.31	−616.54	−33.76	1842.45
	3	Ankle_Left	−1008.72	56.36	1988.34	−922.10	175.26	1918.26	−917.52	160.04	1927.88
	4	Knee_Left	−614.77	88.59	1823.68	−588.03	182.16	1832.79	−585.62	173.24	1835.99
	5	Pelvis	−171.81	−9.58	1780.61	−211.96	69.96	1762.81	−209.90	68.89	1759.34
	6	Spine_Naval	21.34	−9.79	1729.99	102.54	37.97	1707.32	104.79	39.16	1706.74
	7	Hip_Right	−175.64	−100.41	1766.37	−209.87	−14.81	1759.38	−207.30	−16.10	1755.45
	8	Hip_Left	−167.56	91.15	1796.40	−196.20	143.03	1778.89	−194.73	142.01	1776.99
	9	Wrist_Left	263.87	726.84	1725.54	297.23	742.99	1823.48	288.25	742.88	1812.57
	10	Elbow_Left	321.61	474.87	1748.98	327.25	491.17	1800.85	326.41	494.07	1795.62
	11	Wrist_Right	139.72	−711.43	1628.11	197.53	−648.90	1740.66	181.54	−641.17	1755.08
	12	Elbow_Right	298.69	−508.78	1680.13	274.45	−399.28	1748.29	282.42	−403.92	1744.10
	13	Shoulder_Right	342.82	−197.11	1665.62	382.95	−120.35	1755.42	381.05	−117.76	1754.38
	14	Neck	418.65	−17.57	1666.19	434.28	30.41	1748.57	437.31	32.63	1751.23
	15	Spine_Chest	178.09	−11.68	1699.94	225.62	31.75	1694.56	228.14	34.32	1698.10
	16	Shoulder_Left	339.89	175.64	1672.99	383.64	198.84	1755.97	381.10	199.85	1751.66
15°	1	Ankle_Right	−984.67	−121.62	1882.06	−929.25	−26.82	1825.63	−927.29	−34.47	1851.35
	2	Knee_Right	−595.03	−109.03	1707.27	−634.96	−41.66	1777.48	−636.81	−36.43	1774.68
	3	Ankle_Left	−974.33	60.96	1990.39	−930.93	142.59	1893.71	−924.32	146.16	1935.49
	4	Knee_Left	−592.47	83.01	1810.49	−612.19	157.37	1840.44	−606.64	157.57	1837.52
	5	Pelvis	−153.99	−5.12	1751.85	−233.15	78.98	1744.09	−229.19	85.16	1739.42
	6	Spine_Naval	33.73	−1.54	1691.71	90.07	43.08	1685.89	94.77	53.49	1685.95
	7	Hip_Right	−154.85	−95.62	1743.78	−234.93	−1.03	1722.72	−231.39	4.01	1717.35
	8	Hip_Left	−153.04	95.24	1760.79	−219.39	149.73	1772.24	−215.18	156.45	1770.56
	9	Wrist_Left	228.25	722.38	1771.50	240.26	731.07	1880.25	251.22	739.53	1840.89
	10	Elbow_Left	298.19	476.90	1748.38	278.60	480.96	1831.46	286.02	490.25	1818.82
	11	Wrist_Right	273.34	−723.05	1474.17	296.09	−653.51	1632.49	320.39	−629.03	1625.09
	12	Elbow_Right	316.36	−480.41	1555.63	310.44	−409.42	1673.01	350.78	−378.84	1689.04
	13	Shoulder_Right	358.32	−179.00	1620.47	378.35	−116.20	1714.08	377.62	−103.38	1717.54
	14	Neck	424.77	0.88	1623.38	426.06	44.12	1730.31	432.48	56.47	1735.69
	15	Spine_Chest	186.90	0.31	1654.60	215.67	41.38	1672.70	221.02	52.58	1671.88
	16	Shoulder_Left	357.27	190.75	1659.61	381.81	194.42	1747.79	387.72	205.48	1752.88
30°	1	Ankle_Right	−961.56	−127.33	1843.05	−930.58	−13.37	1796.87	−924.88	−14.38	1828.54
	2	Knee_Right	−584.75	−90.11	1672.12	−635.62	−19.11	1747.83	−636.64	−16.27	1742.99
	3	Ankle_Left	−943.11	46.52	1997.76	−930.74	135.16	1904.42	−920.25	142.21	1944.43
	4	Knee_Left	−574.94	85.26	1818.96	−611.52	158.80	1857.32	−605.24	165.05	1856.77
	5	Pelvis	−152.81	−10.48	1724.53	−232.25	102.16	1748.15	−230.40	107.45	1750.23
	6	Spine_Naval	33.21	−2.09	1678.98	91.12	77.99	1684.94	94.33	79.62	1686.50
	7	Hip_Right	−155.77	−94.38	1697.00	−234.66	29.75	1708.09	−234.70	35.17	1709.94
	8	Hip_Left	−149.54	82.55	1755.07	−217.69	163.87	1792.63	−213.86	168.67	1796.54
	9	Wrist_Left	245.88	556.86	1635.89	247.38	697.02	2043.84	258.79	711.82	1912.97
	10	Elbow_Left	286.85	435.28	1849.49	282.86	465.68	1935.11	288.07	464.29	1934.20
	11	Wrist_Right	241.91	−654.73	1367.10	290.47	−587.02	1462.72	316.28	−565.36	1494.09
	12	Elbow_Right	293.88	−425.33	1458.58	306.85	−360.10	1562.81	324.58	−366.68	1579.94
	13	Shoulder_Right	356.87	−151.10	1569.83	377.42	−86.35	1675.26	379.49	−88.08	1675.85
	14	Neck	414.21	18.92	1619.28	427.06	64.93	1731.18	430.84	62.95	1736.03
	15	Spine_Chest	183.30	2.68	1648.34	216.77	78.71	1672.56	220.64	81.01	1672.99
	16	Shoulder_Left	355.78	190.08	1696.86	383.68	206.97	1784.32	390.44	206.70	1791.37
45°	1	Ankle_Right	−988.68	−122.79	1823.42	−931.72	8.07	1767.61	−925.22	7.84	1774.56
	2	Knee_Right	−604.83	−72.16	1657.31	−636.24	11.19	1720.32	−633.59	17.25	1715.18
	3	Ankle_Left	−965.66	6.29	1993.24	−930.76	125.95	1908.22	−919.36	109.26	1939.91
	4	Knee_Left	−588.76	65.84	1824.38	−610.89	157.01	1869.75	−603.10	161.93	1870.89
	5	Pelvis	−164.75	−11.89	1695.88	−231.34	124.34	1752.69	−228.31	129.73	1751.49
	6	Spine_Naval	24.49	1.23	1652.35	92.25	112.53	1687.45	95.75	118.38	1683.65
	7	Hip_Right	−169.19	−89.72	1653.51	−234.20	63.86	1696.64	−232.17	67.97	1698.83
	8	Hip_Left	−159.82	74.42	1742.87	−216.72	173.34	1810.98	−212.65	179.41	1806.20
	9	Wrist_Left	223.74	467.21	1706.84	252.34	625.12	2188.58	258.00	628.87	2177.64
	10	Elbow_Left	302.89	368.26	1921.51	286.44	426.35	2026.10	290.77	430.41	2010.70
	11	Wrist_Right	225.72	−545.64	1161.43	285.49	−481.78	1310.37	287.23	−484.87	1321.89
	12	Elbow_Right	291.07	−379.30	1341.14	303.53	−285.62	1463.11	303.46	−261.94	1466.59
	13	Shoulder_Right	343.78	−133.40	1511.05	377.11	−47.77	1640.17	376.33	−36.74	1635.05
	14	Neck	413.13	14.64	1600.52	427.54	85.08	1731.03	429.60	93.46	1721.73
	15	Spine_Chest	177.98	6.12	1627.35	217.77	115.14	1675.48	221.16	121.32	1671.19
	16	Shoulder_Left	365.38	170.77	1709.64	385.60	210.49	1817.69	391.93	216.10	1807.59

**Table A3:** Cosine similarities calculated from the data in Appendix A Table A1.

Distance	1500 mm		2000 mm		2500 mm		3000 mm		3500 mm		4000 mm	
Orientation	$COS_{H}$	$COS_{fH}$	$COS_{H}$	$COS_{fH}$	$COS_{H}$	$COS_{fH}$	$COS_{H}$	$COS_{fH}$	$COS_{H}$	$COS_{fH}$	$COS_{H}$	$COS_{fH}$
0°	0.4283	0.7327	0.7127	0.9849	0.4168	0.6579	0.7698	0.9214	0.8127	0.9796	0.7872	0.9841

**Table A4:** Cosine similarities calculated from the data in Appendix A Table A2.

Distance	2000 mm		2500 mm		3000 mm		3500 mm		4000 mm	
Orientation	$COS_{H}$	$COS_{fH}$	$COS_{H}$	$COS_{fH}$	$COS_{H}$	$COS_{fH}$	$COS_{H}$	$COS_{fH}$	$COS_{H}$	$COS_{fH}$
−45°	−0.1362	0.9853	−0.0504	0.9845	−0.0363	0.9728	0.5672	0.9507	−0.0368	0.9596
−30°	0.0724	0.8493	0.7436	0.9243	0.7660	0.9375	0.7504	0.9226	0.0286	0.7229
−15°	0.7112	0.9653	0.7313	0.9527	0.7427	0.9732	0.7343	0.9569	−0.0776	0.9753
0°	0.7127	0.9849	0.4168	0.6579	0.7698	0.9214	0.8127	0.9796	0.7872	0.9841
15°	0.7928	0.9726	0.8122	0.9888	0.7561	0.9526	0.6730	0.9290	0.5998	0.9351
30°	−0.1009	0.9770	−0.0833	0.9667	0.1693	0.9267	0.6941	0.9531	0.7011	0.9866
45°	−0.2969	0.8619	0.5375	0.9203	0.2850	0.2859	0.1841	0.6947	0.0205	−0.0486

**Table A5:** Average cosine similarities in dynamic tests.

Test Scenario	Distance (mm)	$COS_{H}$	$COS_{fH}$
Forward and backward walking	-	0.9744	0.9781
Lateral walking	2000	0.9003	0.9162
	2500	0.9762	0.9960
	3000	0.9820	0.9961
Marching in place	2000	0.9770	0.9949
	2500	0.9814	0.9875
In-place body rotation	2000	0.9625	0.9681
